# ﻿*Dryopteris pycnolepis*, a new fern species from northern Vietnam, with miscellaneous notes on *Dryopteris* sect. *Splendentes* (Dryopteridaceae)

**DOI:** 10.3897/phytokeys.263.163351

**Published:** 2025-09-15

**Authors:** Jun-Kai Huang, Xiao-Ji Li, Lian-Xuan Zhou, Zheng-Yu Zuo, Shi-Yong Dong

**Affiliations:** 1 Key Laboratory of National Forestry and Grassland Administration on Plant Conservation and Utilization in Southern China, South China Botanical Garden, Chinese Academy of Sciences, Guangzhou 510650, China South China Botanical Garden, Chinese Academy of Sciences Guangzhou China; 2 University of Chinese Academy of Science, Beijing 100049, China University of Chinese Academy of Science Beijing China; 3 Center for Interdisciplinary Biodiversity Research & College of Forestry, Shandong Agricultural University, Tai’an, 271018, China Shandong Agricultural University Tai’an China

**Keywords:** *Dryopteris splendens*, morphology, phylogeny, plastid genome, taxonomy

## Abstract

A new woodfern species, *Dryopteris pycnolepis*, is described from northern Vietnam. The new species differs mainly in having broadly lanceolate scales (versus ovate-lanceolate) on the stipe and rachis, lateral pinnae with the basal acroscopic pinnules longer than their immediately adjacent pinnules (versus subequal), and fibrillose scales present on both surfaces of pinnules (versus glabrous). Phylogenetic analyses of plastome sequences support both the distinctness of *D. pycnolepis* as a species and its close affinity to *D. sikkimensis*. Morphological comparisons and phylogenetic analyses confirm that these two species, together with *D. rubrobrunnea* and *D. splendens*, form a small natural group (i.e., sect. Splendentes) within the *Dryopteris* clade of this genus. It appears to be a replacement species of the Sino-Himalayan *D. sikkimensis* in northern Vietnam. Section Splendentes is shown to be geographically restricted to the Sino-Himalaya region and northern Vietnam, excluding eastern Asian species previously included in this section. The discrepancy between phylogenetically and morphologically inferred interspecific relationships within sect. Splendentes is highlighted and briefly discussed. The correct authorship of the name *D. splendens* is noted to be “(Bedd.) Kuntze.”

## ﻿Introduction

*Dryopteris* Adans., commonly known as woodferns, is one of the largest genera of ferns, consisting of ca. 348 species (excluding hybrids) (Hassler 1994–2025). This genus is widely distributed in temperate and montane tropical regions of the world, with its distribution center in the Sino-Himalaya region, including western China (SE Xizang, Yunnan, and Sichuan), the eastern Himalaya, and northern Burma (Fraser-Jenkins 1989). According to Fraser-Jenkins (1989), approximately 76 species of *Dryopteris* s. str., excluding species traditionally placed in *Acrophorus* C.Presl, *Acrorumohra* (H.Ito) H.Ito, *Diacalpe* Blume, *Dryopsis* Holttum & P.J.Edwards, *Nothoperanema* (Tagawa) Ching, *Peranema* D.Don, and *Stenolepia* Alderw., are present in this region. All these satellite genera were phylogenetically resolved within *Dryopteris* (Zhang et al. 2012; Kuo et al. 2016) and thus were subsumed under *Dryopteris* taxonomically (PPG I 2016). The broadened *Dryopteris* has been shown to be a monophyletic genus, forming a sister relationship to *Arachniodes* Blume in Dryopteridaceae (Zhang et al. 2012; Zuo et al. 2025), with all sampled species resolved into 13 major clades (Zhang and Zhang 2012). Under the current monophyletic circumscription of *Dryopteris*, the Sino-Himalaya region (distribution center of the genus) harbors 204 species according to the database compiled by Hassler (1994–2025), representing approximately 60% of the species diversity of the whole genus. In contrast, species richness declines dramatically in southern regions adjacent to this center. For instance, only 30 species of *Dryopteris* are listed in Vietnam and 34 species in nearly the whole Indochina (including Thailand but excluding northern Myanmar) (Hassler 1994–2025).

During a botanical expedition to northern Vietnam in 2023, one of the authors (LXZ) collected three specimens of an unknown fern species (voucher number *16451*). The densely scaly, adaxially grooved frond axes and the discrete, round sori with round-reniform indusia indicate that the specimens represent a dryopteroid species. By reviewing the morphology of all described species of Dryopteridaceae in Vietnam and adjacent regions, we confirmed that the unknown species is morphologically most similar to *Dryopteris sikkimensis* (Bedd.) Kuntze, a very distinctive higher-Himalayan forest species (Fraser-Jenkins et al. 2018). *Dryopteris sikkimensis* has been considered closely related to *D. splendens* (Bedd.) Kuntze and *D. rubrobrunnea* W.M.Chu in the Sino-Himalaya region, which, together with a few eastern species, constitute sect. Splendentes Fraser-Jenkins under Dryopteris subg. Dryopteris (Fraser-Jenkins 1986, 1989; Fraser-Jenkins et al. 2018). To determine the identity of the unknown species from northern Vietnam, we conducted morphological comparisons and plastid sequence analyses between this unknown species and members of sect. Splendentes. The results are reported here.

## ﻿Methods

The morphological observation of the unknown species from northern Vietnam is based on herbarium specimens prepared (*LXZ 16451*, three fronds in five sheets) and deposited in the Herbarium of the South China Botanical Garden, Chinese Academy of Sciences (IBSC), as well as on living plants in the wild. Comparisons to species of Dryopteris sect. Splendentes (*D. rubrobrunnea*, *D. splendens*, and *D. sikkimensis*) are based on a thorough examination of specimens from 19 herbaria: B, BR, C, CDBI, E, GH, H, K, KUN, L, MICH, MO, NY, P, PE, S, US, TENN, and ZT. The spore morphology of the unknown species was examined using a JEOL JSM-IT210 scanning electron microscope at 15 kilovolts accelerating voltage in the public laboratory of the South China Botanical Garden, Chinese Academy of Sciences. Prior to observation, samples were sputter-coated with a 10 nm platinum layer using a SuPro Instruments Mini Coater.

To infer the systematic position of the unknown species within *Dryopteris*, we performed sequencing and phylogenetic analysis of the whole plastome. Our sampling strategy included representatives of Dryopteris sect. Splendentes from the Sino-Himalaya region, species related to this section as indicated by previous phylogenetic studies (e.g., Zhang et al. 2012; Kuo et al. 2024; Zuo et al. 2025), and some species collected from Guangdong, southern China, with two to four samples per species to assess intraspecific plastome divergence. The final dataset comprised 40 *Dryopteris* accessions and three outgroup (*Arachniodes*) accessions (Appendix 1). Of the 43 plastomes analyzed, 20 were sourced from NCBI GenBank, and 23 were newly sequenced using silica-gel-dried leaf fragments collected in the field.

For each sample, total DNA was extracted from 15 mg of leaf fragments using the Magnetic Plant Genomic DNA Kit (Tiangen Biotech, Beijing). Libraries were constructed and sequenced (150-bp paired-end reads, >2 Gb per sample) on the MGI DNBSEQ-T7 platform by Anoroda Gene Technology Company (Beijing). Raw reads were assembled into plastomes using GetOrganelle v.1.7.7.0 (Jin et al. 2020), then circularized and annotated in Geneious 9.0.2 (Kearse et al. 2012) with the plastome of *D. rubrobrunnea* (Du et al. 2021) as the reference. These new plastomes and the 20 plastomes from GenBank were aligned with MAFFT v.7 (Katoh and Standley 2013), manually refined in Geneious v.9.0.2 (Kearse et al. 2012), and gap-trimmed using trimAl v.1.4 (Capella-Gutierrez et al. 2009).

The matrix containing 43 plastomes was analyzed using maximum likelihood (ML), maximum parsimony (MP), and Bayesian inference (BI). ML analysis was performed in IQ-TREE v.2.1.4 (Minh et al. 2020) with 10,000 ultrafast bootstrap (UFBoot) replicates to assess branch support. For MP analysis, PAUP* ver. 4.0a169 (Swofford 1991) was employed under equal character weighting, treating gaps as missing data; heuristic searches involved 1,000 replicates using tree bisection-reconnection (TBR) branch swapping, saving 100 trees per random sequence addition, and MP bootstrap values (MPBS) were calculated from 1,000 replicates. Bayesian inference was conducted as follows: the best substitution model was first selected via jModelTest 2.1.10 (Darriba et al. 2012), after which MrBayes 3.2.7 (Ronquist et al. 2012) executed four parallel Markov chain Monte Carlo (MCMC) chains for 10 million generations, sampling one tree every 1,000 generations. Bayesian posterior probabilities (BIPP) were derived from the 75% majority-rule consensus tree after discarding the initial 25% of samples as burn-in.

## ﻿Results

**Figure 1.**
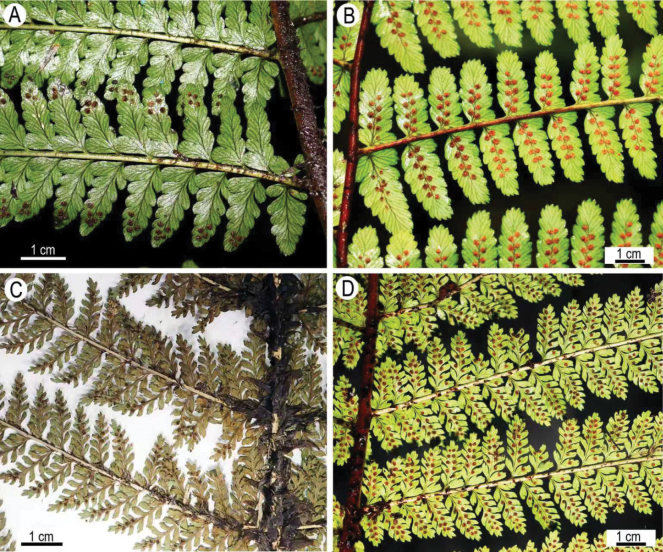
Morphological comparison of four species in *Dryopteris sect. Splendentes*. A. *D. rubrobrunnea* (*Zuo 7158*, KUN); B. *D. splendens* (*Zuo 2740*, KUN); C. *D. pycnolepis* (*Zhou 16451*, IBSC); D. *D. sikkimensis* (*Zuo 2565*, KUN).

The morphological study showed that the specimens (*LXZ 16451*) from northern Vietnam share some key characters with specimens of Dryopteris sect. Splendentes from the Sino-Himalaya region: large bipinnate fronds, less copious scales on the basal stipe, broad scales on the rachis, linear-lanceolate and sessile (rarely very shortly petiolulate) pinnae, and oblong and mostly short-petiolulate pinnules. These are characters distinguishing sect. Splendentes from other groups of *Dryopteris* with non-bullate scales (Dryopteris subg. Dryopteris sensu Fraser-Jenkins). All these specimens exhibit two distinct states of pinnule dissection: lobate (or pinnatifid toward the base) occurring in *D. rubrobrunnea* and *D. splendens* (Fig. 1A, B), and pinnatisect occurring in *D. sikkimensis* and the specimens from northern Vietnam (Fig. 1C, D). Compared with the three other species, the specimens from northern Vietnam are unique in having very densely distributed, broad-lanceolate scales on the stipe (excluding the basal portion) and rachis (Fig. 1C), fibrillose scales on both surfaces of pinnules, and auriculate acroscopic bases of pinnae (Table 1). Our morphological observations indicate that the specimens (*LXZ 16451*) from northern Vietnam represent a distinct species, which we herein call *D. pycnolepis*.

**Table 1.** Morphological comparison of four species in Dryopteris sect. Splendentes.

Character	*D. rubrobrunnea*	*D. splendens*	*D. pycnolepis*	*D. sikkimensis*
Color of stipe & rachis	Reddish brown or occasionally stramineous	Atrocastaneous*	Stramineous	Stramineous or occasionally reddish brown
Scale abundance on stipe & rachis	Sparse or somewhat dense	Sparse	**Very dense**	Sparse or sometimes dense
Large scale on stipe & rachis	Ovate or Ovate-lanceolate	Ovate-lanceolate	**Broadly lanceolate**	Ovate-lanceolate
Lamina texture	herbaceous	**Subcoriaceous**	herbaceous	herbaceous
Pinnule orientation	Ascending 60–80° to costa	**Patent or slightly reflexed**	Ascending 50–60° to costa	Ascending 50–70° to costa
Pinnule division	Lobate or pinnatifid toward base	Lobate or pinnatifid toward base	Pinnatisect	Pinnatisect
Basal pinnule on acroscopic costa	Equal in length to the next one	Equal in length to the next one	**Obviously longer than the next one**	Equal in length to the next one
Pinnule surface	Glabrous	Glabrous	**Scaly, scales fibrillose**	Glabrous
Fertile portion of pinnule	Distal half	**Whole length** (except apical portion)	Distal half	Whole length (frequently leaving basal 1–3 lobes sterile)
Sori number per pinnule	2–4(5) pairs	4–8 pairs	2–3 pairs	4–8 pairs
Sori position	**Medial or inframedial**	Costular	Close to costules	Close to costules

*Terms in bold indicate a unique character state for the corresponding species.

In the resultant matrix of 43 plastomes, the aligned sequence length is 139,878 base pairs (bp), of which 23,447 (16.8%) are variable sites and 14,637 (10.5%) are parsimony-informative sites. The length of the most parsimonious tree is 38,029 steps, with a consistency index of 0.72, a retention index of 0.885, and a rescaled consistency index of 0.637. The log-likelihood score of the ML tree is −420,260.758.

**Figure 2.**
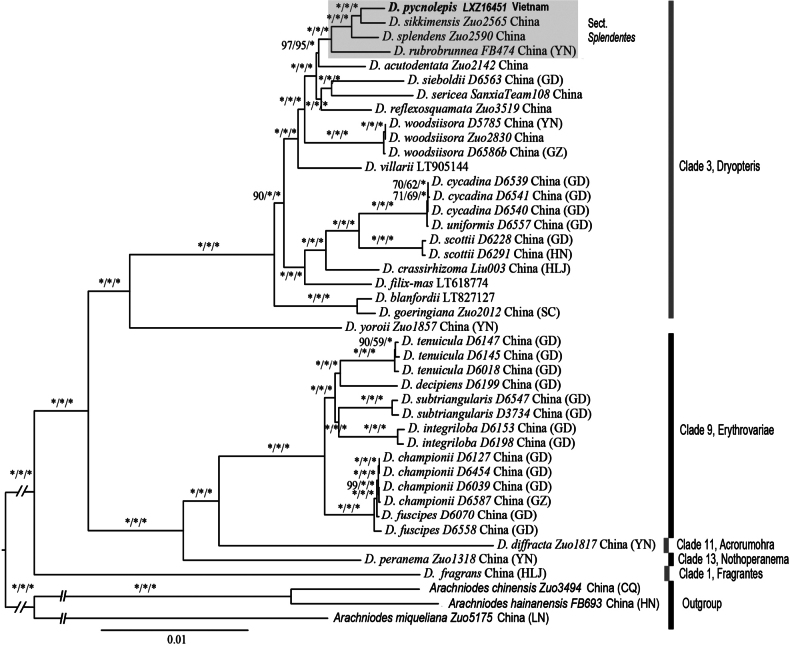
Maximum likelihood phylogeny of selected species of *Dryopteris* based on plastome sequences. Five out of the 13 clades proposed by Zhang et al. (2012) and the previously unsampled Yoroii clade are annotated on the right. The position of sect. Splendentes (four accessions) is highlighted by a grey frame, with an arrow indicating the placement of the new species.

Phylogenetic analyses using ML, MP, and BI produced identical tree topologies, with nearly all branching nodes receiving maximal support values (UFBoot/MPBS = 100%, BIPP = 1.0). All ingroups (specimens of *Dryopteris*) were well resolved into six clades: Fragrantes, Nothoperanema, Acrorumohra, Erythrovariae, Yoroii, and *Dryopteris* (Fig. 2). The Fragrantes formed the most basal clade of the genus, being sister to all other *Dryopteris* species. The three clades – Nothoperanema, Acrorumohra, and Erythrovariae – formed a well-supported group, which was sister to the clades Yoroii and *Dryopteris*. The members of sect. Splendentes, including the new species *D. pycnolepis*, each represented by one accession, were resolved as a strongly supported monophyletic group within the *Dryopteris* clade, forming a sister relationship to *D. acutodentata* Ching. *Dryopteris acutodentata* is also an alpine forest species in the Sino-Himalaya region but differs morphologically from members of sect. Splendentes by its stipe base densely covered with lanceolate scales, 1-pinnate-pinnatifid lamina, and markedly short, triangular-lanceolate pinnae. Within sect. Splendentes, *D. rubrobrunnea* and *D. splendens* successively formed sister clades to the remaining species, with *D. pycnolepis* and *D. sikkimensis* constituting a terminal pair.

The comparison of plastome divergence between *D. pycnolepis* and *D. sikkimensis* with divergence among other conspecific accessions is presented in Table 2. The divergence between *D. pycnolepis* and *D. sikkimensis* measures 454 polymorphic sites (3.25‰ of total length), which is much higher than that observed in three species of the *Dryopteris* clade and four species of the Erythrovariae clade. Intraspecific plastome divergence across these examined accessions ranged from 1 to 111 sites (0.01–0.79‰ of plastome length).

**Table 2.** Plastome divergence between *Dryopteris pycnolepis* and *D. sikkimensis* compared to intraspecific divergence within other *Dryopteris* species.

Species name (accession numbers)	Systematic position	Sequence divergence (sites)	Permillage difference
*Dryopteris pycnolepis* (1)–*D. sikkimensis* (1)	*Dryopteris* clade	454	3.25
*Dryopteris woodsiisora* (3)	*Dryopteris* clade	2–30	0.01–0.21
*Dryopteris cycadina* (3)	*Dryopteris* clade	3–17	0.02–0.12
*Dryopteris scottii* (2)	*Dryopteris* clade	58	0.41
*Dryopteris tenuicula* (3)	*Erythrovariae* clade	65–81	0.46–0.58
*Dryopteris subtriangularis* (2)	*Erythrovariae* clade	112	0.80
*Dryopteris integriloba* (2)	*Erythrovariae* clade	117	0.84
*Dryopteris championii* (4)	*Erythrovariae* clade	1–27	0.01–0.19
*Dryopteris fuscipes* (2)	*Erythrovariae* clade	111	0.79

## ﻿Discussion

**Figure 3.**
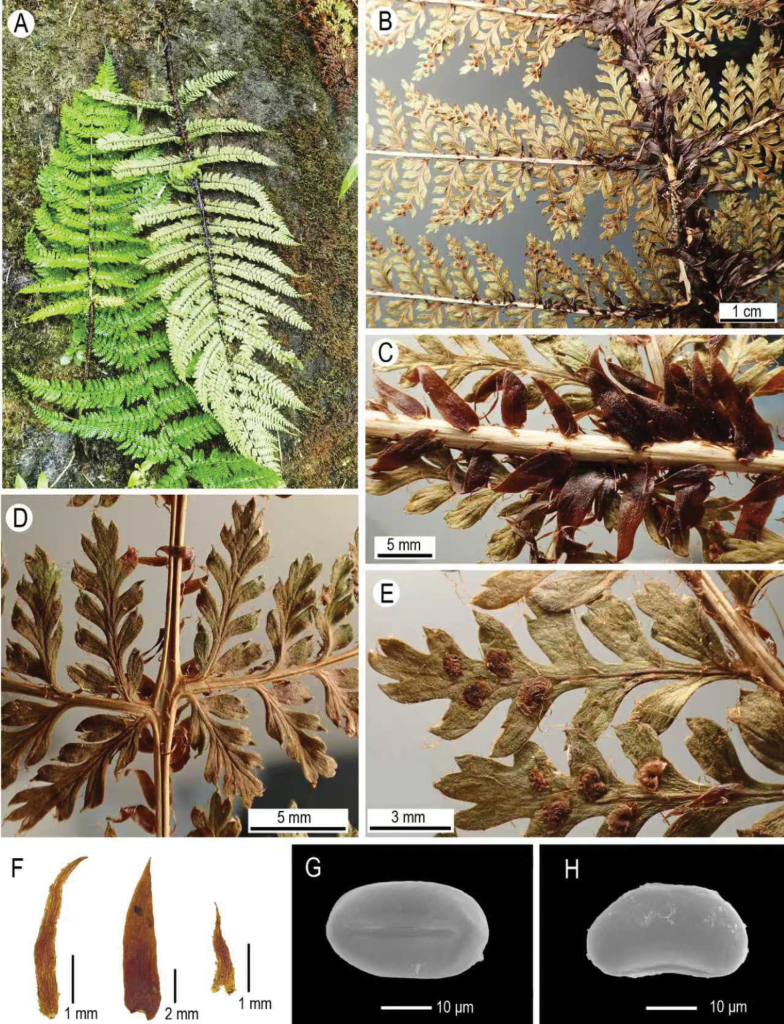
Morphology of *Dryopteris pycnolepis* Z.Y.Zuo & S.Y.Dong. A. Three fronds cut in the field; B. Three pairs of middle pinnae on rachis (abaxial view); C. Detail of rachis scales (abaxial view); D. Detail of a pair of upper pinnae (basal portions) on rachis (adaxial view); E. Detail of two pinnules on a pinna (abaxial view), showing fibrillose scales on pinnule surface and sori on distal half of pinnules; F. Scales on stipe; G. Proximal view of a spore; H. Lateral view of a spore. (All from *L.X.Zhou 16451* at IBSC).

Both morphological comparisons and phylogenetic analyses support that the specimens (*LXZ 16451*) from northern Vietnam represent a distinct species (*Dryopteris pycnolepis*). The markedly densely distributed scales on the stipe and rachis characterize *D. pycnolepis* (Figs 1C, 3B, C), clearly distinguishing this species from congeners with similar frond morphology. Additional diagnostic features separating *D. pycnolepis* from its closest relative, *D. sikkimensis*, include broadly lanceolate scales (versus ovate-lanceolate), the basal acroscopic pinnule longer than its adjacent pinnules (versus subequal), and fibrillose scales present on both surfaces of pinnules (versus glabrous). Morphological characters regarding scale shape and position on fronds, as well as the symmetry of the pinna base, are considered of taxonomic significance and thus are widely used in recognizing different sections within *Dryopteris* (Fraser-Jenkins 1986, 1989; Wu and Lu 2000; Wu et al. 2013). Phylogenetically, there is significant plastome divergence between accessions of *D. pycnolepis* and *D. sikkimensis*, evidenced by long branches in our phylogram (Fig. 2) and by divergence values much higher than observed intraspecific variations (Table 2), lending further support to the distinctness of *D. pycnolepis* as a separate species. Morphological and molecular evidence indicates that *D. pycnolepis* is possibly a replacement species of the Sino-Himalayan *D. sikkimensis* in northern Vietnam.

Both the morphological and phylogenetic studies support *D. pycnolepis*, *D. sikkimensis*, *D. rubrobrunnea*, and *D. splendens* as constituting a small natural group (sect. Splendentes) within *Dryopteris*. According to our observations on herbarium specimens and living plants, this section morphologically differs from other *Dryopteris* species or sections mainly in the less abundant scales on the basal portion of the stipe and the amply bipinnate lamina with sessile, linear-lanceolate pinnae. So far as we know, there are only these four species in sect. Splendentes. When establishing Dryopteris sect. Splendentes, Fraser-Jenkins (1986) assigned *D. bamleriana* Rosenst. (endemic to New Guinea), *D. kwanzanensis* Tagawa (endemic to Taiwan), and *D. reflexosquamata* Hayata (distributed from SW China to Taiwan) to this section along with species in the Sino-Himalaya region. All three species are clearly different from *D. pycnolepis* and other Sino-Himalayan members of sect. Splendentes by having much shorter pinnae (not linear-lanceolate). In addition, *D. bamleriana* differs in having obviously petiolulate pinnae (5–8 mm long), while both *D. kwanzanensis* and *D. reflexosquamata* differ by having the stipe base covered with abundant, reflexed scales. Phylogenetic analyses indicate that *D. reflexosquamata* and *D. kwanzanensis* are not members of sect. Splendentes. As shown in Fig. 2, the sampled *D. reflexosquamata* was resolved in a subclade with *D. sieboldii* and *D. sericea* within the *Dryopteris* clade, while *D. kwanzanensis* was demonstrated to be closely allied to *D. reflexosquamata* (Kuo et al. 2024). Thus, we conclude that Dryopteris sect. Splendentes is a small natural group restricted to the Sino-Himalaya region and northern Vietnam, currently comprising only four species with large-sized, bipinnate fronds, less abundant scales on the basal stipe, and sessile, linear-lanceolate pinnae.

Within Dryopteris sect. Splendentes, the interspecific relationships indicated by morphological comparison are not fully consistent with those suggested by plastome-based phylogenetic analyses. Except for the sister relationship between *D. pycnolepis* and *D. sikkimensis*, the relationships among *D. rubrobrunnea*, *D. splendens*, and the *D. pycnolepis*–*D. sikkimensis* clade differ between morphological and molecular evidence. Our morphological observations show that *D. rubrobrunnea* is very similar to *D. splendens*, both having nearly the same pattern of frond dissection (lobate or pinnatifid toward the base) (Figs 1A, B). *Dryopteris rubrobrunnea* (originally recorded as *D. rubripes* Ching & Chu, ined.) is morphologically intermediate between *D. splendens* and *D. sikkimensis* and is nearer to the former (Fraser-Jenkins 1989: 407, 408). Indeed, *D. splendens* possesses some unique characters, such as subcoriaceous lamina texture (versus herbaceous), patent or more-or-less reflexed pinnules (versus ascending), and sori borne on pinnules from the base to nearly the apex (versus restricted to the distal half of the pinnule) (Table 1), which make it the most distinct within sect. Splendentes. However, our phylogenetic analyses neither support the closest affinity of *D. rubrobrunnea* to *D. splendens* nor support that *D. rubrobrunnea* is closer to *D. splendens* than to the *D. pycnolepis*–*D. sikkimensis* clade (Fig. 2). The phylogenetic relationships indicating that *D. splendens* is closer to *D. pycnolepis* and *D. sikkimensis* than to *D. rubrobrunnea* are currently difficult to interpret morphologically. It must be acknowledged that the morphology of species in sect. Splendentes remains insufficiently understood. For example, the spore morphology of *D. pycnolepis* appears unique (with smooth and glabrous perispores; Fig. 3F, G) when compared to the richly ornamented perispores in other species of *Dryopteris* (Tryon and Lugardon 1991; Wang and Dai 2010). However, the spore morphology of all species in sect. Splendentes (except *D. pycnolepis* described here) has not been documented, and spores of these species were unavailable for study. To better understand the morphology of this group, further field observations and the collection of complete fronds with rhizomes and mature spores are needed.

## ﻿Taxonomic treatment

### ﻿ Dryopteris pycnolepis

Taxon classificationPlantaePolypodialesDryopteridaceae

Z.Y.Zuo & S.Y.Dong sp. nov.

F4D4FA4A-0F1C-54B2-B5B2-332871C51B3D

urn:lsid:ipni.org:names:77369252-1

Figs 1C, 3, 4

#### Type.

Vietnam • Lào Cai Province: Sa Pa District, Mt. Hoàng Liên; 22°18.47′N, 103°46.45′E; 17 June 2023; *L.X. Zhou 16451* (holotype, IBSC; isotypes: IBSC).

**Figure 4.**
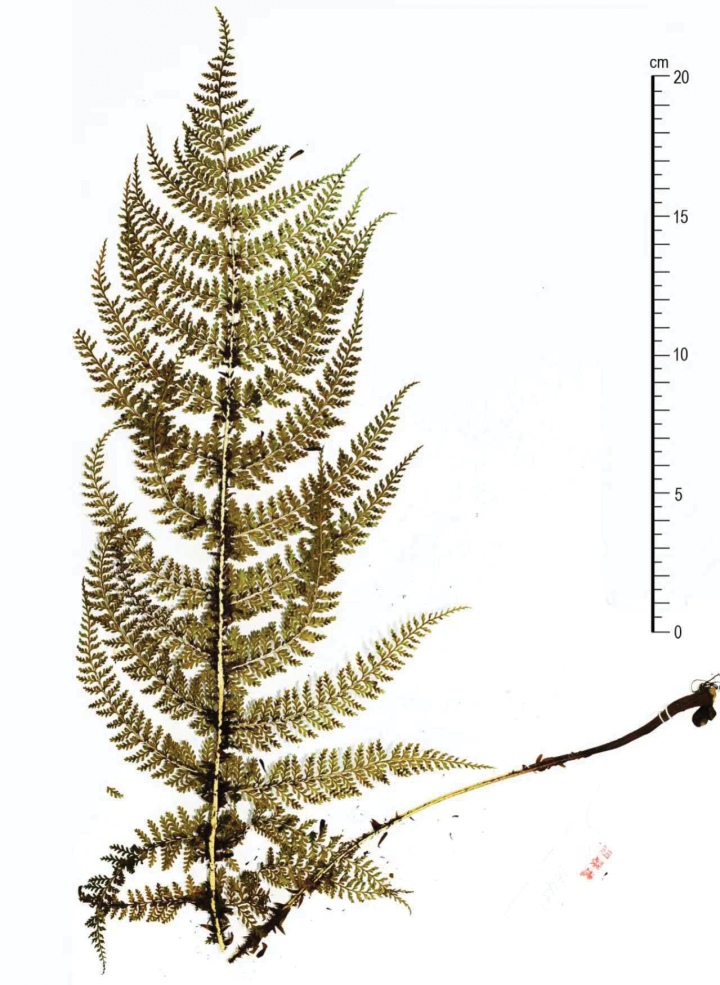
Holotype of *Dryopteris pycnolepis* Z.Y.Zuo & S.Y.Dong (*L.X.Zhou 16451*, IBSC).

#### Diagnosis.

*Dryopteris pycnolepis* is very similar to *D. sikkimensis* but differs in its stipe and rachis very densely covered with large scales (versus obviously sparse, scattered scales) and the pinnules abaxially with long, fibrillose scales (versus pinnules glabrous).

#### Description.

***Plants*** medium to large, 50–90 cm tall. ***Rhizome*** short, with fronds clustered on its apex; ***stipe*** 20–35 cm long (shorter than rachis), 2–3.5 mm thick, thickened at base (to 5 mm), lower portion (in basal 6–8 cm length from the base) dark brown, upwards stramineous, matte, together with rachis narrowly grooved adaxially and densely scaly except for the basal portion in dark brown (which bearing relatively few scales); ***scales*** dark brown or mostly blackish, lustrous, chartaceous, spreading acroscopically or laterally, polymorphic in size, smaller ones lanceolate, ca. 1.2 × 0.2 mm, larger ones or broadly lanceolate, 8–10 × 1.5–2.2 mm, apex acuminate or acute, base truncate or slightly cordate, margins entire or minutely erose; ***lamina*** herbaceous, adaxially dark green and abaxially pale green when living, oblong-lanceolate, 30–55 × 15–25 cm, bipinnate-pinnatisect, apex somewhat abruptly narrowed and acuminate, bearing 13–15 pairs of pinnae below the abruptly reduced terminal portion; ***rachis*** stramineous, densely scaly, with similar scales abaxially and linear or fibrillose scales (to 3–5 mm long) adaxially (the latter borne on rachis groove); ***pinnae*** linear or narrowly linear-lanceolate, spaced 5–8 mm apart except at base (where contiguous), subopposite or sometime those on upper rachis alternative, mostly patently spreading (with an approximately right anger to rachis) at base and distally curved toward frond apex, sessile, all pinnae (except those on terminal reduced portion of lamina) almost of the same size, with the lowest 1–2 pairs not or slightly shortened, middle pinnae 10–15 × 2–3 cm (with the widest base up to 4 cm wide), base asymmetrical, acroscopic base truncate (parallel to rachis and partly overlapping rachis), basiscopic base round or cuneate, apex acuminate, with copious, blackish, lanceolate scales on abaxial costae, the large pinna bearing 15–25 pairs of pinnules; ***pinnules*** generally alternative, obliquely spreading (forming an angle of ca. 50–60° to costa), nearly oblong, very shortly stalked, middle pinnules 1.8–2 cm long and 6–8 mm wide at base, acroscopic base truncate, basiscopic base round, apex obtuse, the acroscopic basal pinnule the longest (to 2.5 cm) and obviously longer than the second pinnule, pinnatisect, with fibrillose scales (ca. 5 mm long) on abaxial surfaces of costules and veins (fewer fibrillose scales present on adaxial surface); lobes of a middle pinnule 5–7 pairs, ascending, narrowly oblong, ca. 2.5–4.5 × 1.2–1.5 mm, apex round or obtuse, apiculate with 1–2 teeth, spaced ca. 1 mm apart; ***veins*** free, slightly visible on both surfaces, simple, once or twice forked, not reaching lamina margins. ***Sori*** round, confined to upper half of pinnules, 2–3 pairs per pinnule, one per segment, dorsal on the base of acroscopic veinlet, close to costules; ***indusia*** orbicular-reniform, large, ca. 1 mm in diameter, approximate to each other, margin entire, brown, membranous, persistent; ***spore*** reniform, monolete, ca. 32 × 16.5–18 µm, with smooth, glabrous surface of perispores.

#### Habitat.

Terrestrial alongside a trail in montane broadleaved evergreen forest, elevation 2860 m; currently only a small population observed in Mt. Hoàng Liên, northern Vietnam.

#### Etymology.

The specific epithet comes from the Latin words “*pycno*-”(densely bearing) and “*lepis*” (scales), referring to the scales densely borne on the stipe, and rachis.

### ﻿ Dryopteris splendens

Taxon classificationPlantaePolypodialesDryopteridaceae

(Bedd.) Kuntze, Revis. Gen. Pl. 2: 813. 1891.

25854CEE-2EE9-5B54-804B-95D7750F6E63

Nephrodium splendens Hook.; Sp. Fil. [W.J.Hooker] 4: 126 (1862), nom. illeg., non Nephrodium splendens (Willd.) Desv. (1827); Lastrea splendens Bedd., Ferns Brit. India 1: t. 42. 1865, nom. nov. Type: INDIA. West Bengal, “Sikkim”; *J.D.Hooker s.n.* (lectotype, K, designated by Fraser-Jenkins in Bull. Brit. Mus. Nat. Hist., Bot. 18(5): 405. 1989, not seen; isolectotypes: B, barcode B200067565, B200067569, B200067570, B200067571; BR, barcode BR0000032264388, BR0000032264395; E, barcode E01467890, E01467891; GH, barcode 00021663; NY, barcode 04153316, 04153317; P, barcode P01603131, P01603132, P01603131; U barcode U.1009785; ZT, barcode ZT-00278799).
Aspidium splendens Willd., Sp. Pl., ed. 4 [Willdenow] 5: 220 (1810). Nephrodium splendens (Willd.) Desv., Mém. Soc. Linn. Paris 6(3): 253 (1827). Type: Mauritius. *Wallich list no. 2241* (holotype, K001115453) (Excluding).

#### Note.

The correct authorship for *Dryopteris splendens* is “(Bedd.) Kuntze,” not “(Hook.) Kuntze” as cited by many authors (e.g., Fraser-Jenkins 1986, 1989; Wu and Lu 2000; Wu et al. 2013; Fraser-Jenkins et al. 2018), nor “(Desv.) Kuntze” as shown in Hassler’s (1994–2025) World Plants database. This taxon was originally described by Hooker (1862) as *Nephrodium splendens*. Hooker’s name, however, is a later homonym of *Nephrodium splendens* (Willd.) Desv. (Desvaux 1827) and is therefore illegitimate. Consequently, it cannot serve as the basonym for subsequent combinations. The second earliest name of this taxon, *Lastrea splendens* Bedd., is legitimate under ICN Art. 6.5 and can be considered, under ICN Art. 6.13, a new name. Therefore, it can serve as the basonym for later combinations where necessary. Thus, the authorship of *Dryopteris splendens* proposed by Kuntze (1891) should be attributed to “(Bedd.) Kuntze.”

## Supplementary Material

XML Treatment for Dryopteris pycnolepis

XML Treatment for Dryopteris splendens

## ﻿Additional information

### Use of AI

No use of AI was reported.

### Author ORCIDs

Jun-Kai Huang https://orcid.org/0009-0003-1497-8173

Zheng-Yu Zuo https://orcid.org/0000-0002-8334-6132

Shi-Yong Dong https://orcid.org/0000-0002-8449-7856

### Data availability

All data are available within the paper. New nucleic acid sequences are being deposited in GenBank.

## ﻿Appendix 1

Accessions were used for phylogenetic analyses in this study. Information is arranged in this order: species name, voucher specimen (collector, number, and herbarium), place of origin, and GenBank number of plastome. The dash (–) indicates information not available; the asterisk (*) indicates sequences newly generated in this study.

***Arachniodes chinensis*** (Rosenst.) Ching, *Z.Y. Zuo 3494* (KUN), China (Chongqing), OR530038. ***Arachniodes hainanensis*** (Ching) Ching, *Q. Wei et al. FB693* (KUN), China (Hainan), MT130572. ***Arachniodes miqueliana*** (Maxim. ex Franch. & Sav.) Ohwi, *Z.Y. Zuo 5175* (KUN), China (Liaoning), OR530039. ***Dryopteris acutodentata*** Ching, *Z.Y. Zuo 2142* (KUN), China (Yunnan), OQ649801. ***Dryopteris blanfordii*** (C. Hope) C. Chr., –, Russia (in cult.), LT827127. ***Dryopteris championii*** (Benth.) C.Chr. ex Ching, *S.Y. Dong 6039* (IBSC), China (Guangdong), PV868338*; *S.Y. Dong 6127* (IBSC), China (Guangdong), PV868339*; *S.Y. Dong 6454* (IBSC), China (Guangdong), PV868340*; *S.Y. Dong 6526* (IBSC), China (Guangdong), PV868341*. ***Dryopteris crassirhizoma*** Nakai, *LiuBD003* (KUN), China (Heilongjiang), MT130689. ***Dryopteris cycadina*** (Franch. & Sav.) C. Chr., *S.Y. Dong 6539* (IBSC), China (Guangdong), PV868342*; *S.Y. Dong 6540* (IBSC), China (Guangdong), PV868343*; *S.Y. Dong 6541* (IBSC), China (Guangdong), PV868344*. ***Dryopteris decipiens*** (Hook.) Kuntze, *7333* (PE), China (Guizhou), KY427348. ***Dryopteris diffracta*** (Baker) C. Chr., *Z.Y. Zuo 1817* (KUN), China (Yunnan), OQ649848. ***Dryopteris filix-mas*** (L.) Schott, –, Russia (in cult.), LT618774. ***Dryopteris fragrans*** (L.) Schott, –, China (Heilongjiang), KX418656. ***Dryopteris fuscipes*** C. Chr., *S.Y. Dong 6070* (IBSC), China (Guangdong), PV868345*; *S.Y. Dong 6558* (IBSC), China (Guangdong), PV868346*. ***Dryopteris goeringiana*** (Kunze) Koidz., *Z.Y. Zuo 2012* (KUN), China (Sichuan), OQ649863. ***Dryopteris integriloba*** C. Chr., *S.Y. Dong 6153* (IBSC), China (Guangdong), PV868347*; *S.Y. Dong 6198* (IBSC), China (Guangdong), PV868348*. ***Dryopteris peranema*** Li Bing Zhang, *Z.Y. Zuo 1318* (KUN), China (Yunnan), OQ649938. ***Dryopteris pycnolepis*** Z.Y.Zuo & S.Y.Dong, *L.X. Zhou 16451* (IBSC), Vietnam, PV868360*. ***Dryopteris reflexosquamata*** Hayata, *Z.Y. Zuo 3519* (KUN), China (Guizhou), OQ649953. ***Dryopteris rubrobrunnea*** W. M. Chu, *Cheng X. et al. FB474* (KUN), China (Yunnan), MT130582. ***Dryopteris scottii*** (Bedd.) Ching, *S.Y. Dong 6228* (IBSC), China (Guangdong), PV868349*; *S.Y. Dong 6291* (IBSC), China (Hainan), PV868350*. ***Dryopteris sericea*** C. Chr., *SanxiaTeam 108* (PE), China (Chongqing), OQ649963. ***Dryopteris sieboldii*** (T. Moore) Kuntze, *S.Y. Dong 6563* (IBSC) China (Hainan), PV868351*. ***Dryopteris sikkimensis*** (Bedd.) Kuntze, *Z.Y. Zuo 2565* (KUN), China (Xizang), OQ649967. ***Dryopteris splendens*** (Hook.) Kuntze, *Z.Y. Zuo 2590* (KUN), China (Xizang), OQ649972. ***Dryopteris subtriangularis*** (C. Hope) C. Chr., *S.Y. Dong 3734* (IBSC), China (Guangdong), PV868352*; *S.Y. Dong 6547* (IBSC), China (Guangdong), PV868353*. ***Dryopteris tenuicula*** Matthew & Christ, *S.Y. Dong 6018* (IBSC), China (Guangdong), PV868354*; *S.Y. Dong 6145* (IBSC), China (Guangdong), PV868355*; *S.Y. Dong 6147* (IBSC), China (Guangdong), PV868356*. ***Dryopteris uniformis*** Makino, *S.Y. Dong 6557* (IBSC), China (Guangdong), PV868357*. ***Dryopteris villarii*** (Bellardi) Woyn. ex Schinz & Thell., –, Russia (in cult.), LT905144. ***Dryopteris woodsiisora*** Hayata, *Z.Y. Zuo 2830* (KUN), China (Yunnan), OQ649996; *S.Y. Dong 5785* (IBSC), China (Yunnan), PV868358*; *S.Y. Dong 6586b* (IBSC), China (Guizhou), PV868359*. ***Dryopteris yoroii*** Seriz., *Z.Y. Zuo 1857* (KUN), China (Yunnan), MW796579.
